# Absenteeism in child health services: a systematic review

**DOI:** 10.1590/0034-7167-2021-0805

**Published:** 2023-05-29

**Authors:** Bianca Machado Cruz Shibukawa, Gabrieli Patrício Rissi, Roberta Tognollo Borotta Uema, Marcela Demitto Furtado, Maria de Fátima Garcia Lopes Merino, Ieda Harumi Higarashi

**Affiliations:** I Universidade Estadual de Maringá. Maringá, Paraná, Brazil

**Keywords:** Absenteeism, Child Health Services, Systematic Review, Child Care, Barriers to Access of Health Services., Absentismo, Servicios de Salud del Niño, Revisión Sistemática, Cuidado del Niño, Barreras de Acceso a los Servicios de Salud., Absenteísmo, Serviços de Saúde da Criança, Revisão Sistemática, Cuidado da Criança, Barreiras ao Acesso aos Cuidados de Saúde.

## Abstract

**Objectives:**

to analyze data from qualitative studies related to the phenomenon of health follow-up dropout of newborns, infants and preschoolers in child health services.

**Methods:**

systematic review, carried out in 19 information bases. Studies were included that portray the reasons for dropping out health follow-up of children up to five years old. The JBI methodology was used for systematic reviews of qualitative evidence.

**Results:**

we identified 20,199 studies. After applying the eligibility criteria, 81 were selected. Seven were excluded due to duplicity, resulting in 74 articles that were read in full. After this phase, three articles were selected for the final sample and later after reading their references, one more was included, totaling four articles for critical analysis.

**Conclusions:**

the synthesized findings highlight that health follow-up dropout is based on personal knowledge and beliefs, the family routine dynamics and access to services.

## RESUMO

**Objetivos:**

analisar dados provenientes de estudos qualitativos relacionados ao fenômeno do abandono do acompanhamento de saúde de recém-nascidos, lactentes e pré-escolares em serviços de saúde criança.

**Métodos:**

revisão sistemática, realizada em 19 bases de informações. Foram incluídas pesquisas que retratam as razões de abandono de acompanhamento de saúde de crianças de até cinco anos. Utilizou-se a metodologia do JBI para revisões sistemáticas de evidências qualitativas.

**Resultados:**

identificaram-se 20.199 estudos. Após a aplicação dos critérios de elegibilidade, 81 foram selecionados. Sete foram excluídos por duplicidade, resultando em 74 artigos que foram lidos na íntegra. Após essa fase, três artigos foram selecionados para amostra final e, posteriormente, após leitura de suas referências, incluiu-se mais um, totalizando quatro artigos para análise crítica.

**Conclusões:**

as descobertas sintetizadas destacam que o abandono do acompanhamento de saúde está alicerçado aos saberes e crenças pessoais, à dinâmica da rotina familiar e ao acesso aos serviços.

## RESUMEN

**Objetivos:**

analizar datos de estudios cualitativos relacionados con el fenómeno del abandono del seguimiento de la salud de los recién nacidos, lactantes y preescolares en los servicios de salud del niño.

**Métodos:**

revisión sistemática, realizada en 19 bases de datos. Se incluyeron estudios que retratan las razones del abandono del seguimiento de la salud de los niños hasta los cinco años. Se utilizó la metodología JBI para revisiones sistemáticas de evidencia cualitativa.

**Resultados:**

se identificaron 20.199 estudios. Después de aplicar los criterios de elegibilidad, se seleccionaron 81. Siete fueron excluidos por duplicidad, resultando en 74 artículos que fueron leídos en su totalidad. Después de esta fase, se seleccionaron tres artículos para la muestra final y, posteriormente, después de la lectura de sus referencias, se incluyó uno más, totalizando cuatro artículos para análisis crítico.

**Conclusiones:**

los hallazgos sintetizados destacan que el abandono del seguimiento de la salud se basa en los conocimientos y creencias personales, la dinámica de la rutina familiar y el acceso a los servicios.

## INTRODUCTION

Child health is the subject of debates at an international level, given the importance of promoting, protecting and recovering the health of this part of the population that will be responsible for the future of the nation^(1-3)^. In 2000, the World Health Organization launched eight millennium goals, among which one was on reducing child mortality. Since then, 189 countries have implemented measures to improve health services for this population^(2-3)^.

Investment in quality programs, combined with good child support in early childhood, in addition to reflecting a positive return for society, it is one of the best strategies to reduce inequalities, face poverty and help build a social environment with more sustainable environmental and psychological conditions^(4)^.

It is estimated that more than 200 million children under the age of five living in developing countries do not reach their potential, due to being exposed to several risk factors, such as environmental, biological and psychosocial, situations that can be evidenced and even remedied, when there is adequate follow-up^(4)^.

According to the Brazilian National Policy for Comprehensive Child Health Care (PNAISC - *Política Nacional de Atenção Integral à Saúde da Criança*), early childhood development should be emphasized in Primary Care, including actions to support families, aimed at training and strengthening bonds between professionals and family members^(5)^.

It is paramount that health professionals, through early contact with children and families, build strategies to encourage health promotion, adequate nutrition and appropriate early stimulation, ensuring that all children reach their development potential^(6)^.

It should also be noted that such employees should provide guidance to parents and families of children in relation to immunization, child growth and development, in addition to highlighting the importance of follow-up in the health service, since the early identification of signs and symptoms can prevent future worsening of diseases^(7)^.

However, in the context of the pandemic experienced, there are studies that demonstrate a decrease in the supply of services offered by primary care, reaching the point of interrupting the provision of care to the child population. Concomitant with this fact, demand from parents and guardians for services has also decreased, as the fear of contamination by COVID-19 still persists. As a result of this situation, there is a decrease in the number of children followed up, delays in the vaccination schedule and lack of regular care^(8-9)^.

In this scenario, nursing plays a crucial role, as it can help in the development of actions that involve child growth and development, together with the reduction of health inequalities so that children not only survive, but also do so with dignity^(10-11)^.

It should be noted that the non-attendance and dropout rates vary between the different levels of health complexity; however, some associated factors are predictive of absence, such as delay in professional care due to overbooking scheduling systems, geographic distances between residence and consultation location, economic factors and satisfaction with the health service^(12)^.

In some situations, families end up choosing to purchase a health insurance, especially when children have special needs and the link with health units close to their home is fragile and ineffective from a family point of view^(13)^.

Absences in low-complexity consultations and even in those that assist children with complex medical conditions are high, with a non-attendance rate of up to 69.4%. The family is the one who has the power to decide whether to continue or interrupt children’s health follow-up. In a study carried out in the United States, of the 39.5% cases that dropped out the child health service, 33.3% were due to family reasons^(14)^.

Considering the importance of compliance with child followup and treatment services and the high rates of dropout that still exist, this study is justified, since in order to put into practice the guidelines of programs related to child health, it is necessary that such care actually takes place. When this is absent, it can cause delays and queues in the health system, unnecessary hospitalizations and, in particular, family and infant suffering due to both the illness and other conditions that would be resolvable if there was follow-up^(11)^.

A preliminary search of PROSPERO, MEDLINE, the Cochrane Database of Systematic Reviews and the JBI was carried out on the subject, no current or ongoing systematic review was identified on the topic in question. Therefore, the data found in this study may guide the practice in child health services, in order to minimize follow-up dropout.

## OBJECTIVES

To analyze data from qualitative studies related to the phenomenon of health follow-up dropout of newborns, infants and preschoolers in child health services.

## METHODS

### Ethical aspects

Since this is a systematic review study, without the involvement of human beings, there was no need for assessment by the Research Ethics Committee.

### Study design, period, and place

This is a systematic review conducted in accordance with the JBI methodology for systematic reviews of qualitative evidence^(15)^, which adopted the following guiding question for this research, in accordance with the PICo strategy^(15)^: what are the reasons given by family members for not taking children to follow-up on growth and development in child health services?

Searches in information sources, such as Web of Science, Science Direct, CINAHL, Scopus, BDENF, PubMed, Embase, LILACS, ASSIA, Sociological Abstracts, OpenGrey, Google Scholar, Darte, Cybertesis, Open Thesis, PeerJ Prepint, MedRxiv, BioRxiv and PsycINFO, took place from November 2020 to March 2021.

The process of identifying articles until the final sample was synthesized and presented following the Preferred Reporting Items for Systematic Reviews and Meta-Analyses (PRISMA)^(16)^.

### Population or sample, inclusion and exclusion criteria

Primary studies focusing on qualitative data, without delimitation of language and year of publication, in which the population referred to family members of children under five years of age, who described the reasons given for dropping out the follow-up of child growth and development in health services children, regardless of the level of care, were included. Studies that were unavailable in full, however, it should be noted that this selection criterion was applied after the corresponding author was contacted and the authors did not receive a response to the request were excluded.

Descriptors were selected from the Health Sciences Descriptors (DeCS) and Medical Subject Headings (MeSH) platforms. The following controlled descriptors in Portuguese and English were listed*: Serviços de Saúde da Criança*/ Child Health Services; *Pacientes não comparecentes*/No-Show Patients; *Pacientes Desistentes do Tratamento*/Patient Dropouts.

Uncontrolled descriptors in Portuguese and English were also included: *Serviços de Neonatologia*/Child Health Service; *Serviços de Saúde do Lactente*/Child Services, Health; *Serviços de Saúde do Recém-Nascido*/Health Service, Child; *Serviços de Saúde Infantil*/Health Service, Infant; *Serviços de Saúde Neonatal*/Health Services, Child; *Pacientes Ausentes*/No Show Patients; *Pacientes Faltantes*/No-Show Patient; *Pacientes que não Comparecem*/No-Show, Patient; *Desistência ao Tratamento*/Non-Attendance, Patient; *Desistência do Paciente*/ Dropout, Patient; *Desistência do Paciente ao Tratamento*/Patient Non-Attendance; *Pacientes que Abandonam o Tratamento*/Patient Dropout; *Pacientes que Desistem do Tratamento*/Dropouts, Patient.

### Study protocol

The search strategy was guided by the librarian at the *Universidade de São Paulo* Nursing School, through the use of Boolean operators. Titles and abstracts were screened by two independent reviewers for assessment based on inclusion criteria. Potentially relevant studies studies, with a level of credibility for each finding; 2- developing categories for sufficiently similar findings; 3- development of one or more findings synthesized from the categories.

Qualitative research findings were grouped using JBI SUMARI with meta-aggregation approach^(15)^, gathering and categorizing information based on similarity in meaning. These categories were further synthesized in order to produce a single comprehensive set of results that can be used as a basis for evidence-based practice.

The final synthesized results were classified according to the ConQual approach to establish confidence in the outcome of the qualitative research synthesis and presented in a synthesis of results^(17)^. The review was registered and approved by PROSPERO under Opinion number CRD42020193702.

## RESULTS

After crossing the descriptors, 20,199 studies were identified, whose titles and abstracts were read. After applying the eligibility criteria, 81 articles were selected. However, seven were excluded due to duplicity, resulting in 74 articles that were read in full. Of these, one was excluded due to lack of access to the original document, even after several attempts to contact the author and the institution responsible for the study. As it is a doctoral thesis defended in 1996, it was not available in digital media.

**Figure 1:**
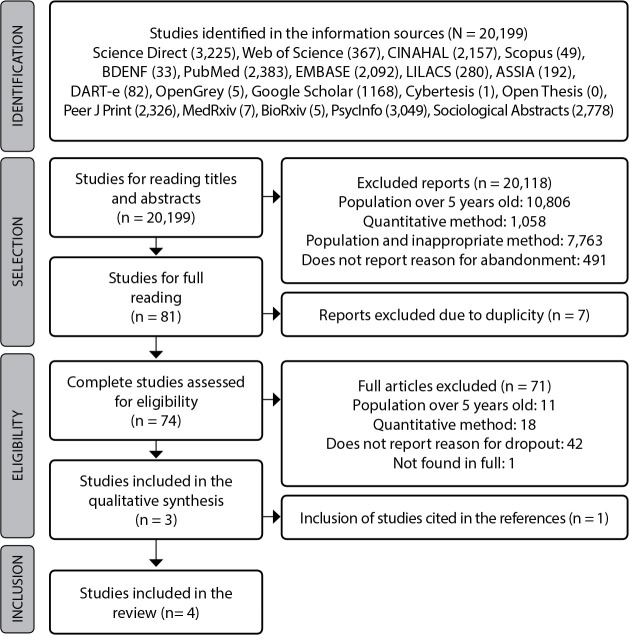
PRISMA flowchart of the study selection process^(16)^

Article references in the final sample were examined in order to identify other studies that could respond to the objective of this research. In this process, one study became eligible. Therefore, four articles were submitted to critical analysis. Figure 1 shows details of this entire process.

After this stage, a critical assessment of qualitative study methodological quality was carried out following the criteria proposed by the JBI. Chart 1 shows the results of this assessment used in the final sample, in which the assessed criteria were exposed and what was identified in each study.

**Chart 1:** Assessment of methodological quality of qualitative studies included in the review, according to the JBI critical assessment instrument^(15)^, Maringá, Paraná, Brazil, 2021

Question	Dias	Madeira	Ximenes-Neto et al	Rechel et al
1. Is there congruence between the philosophical perspective and the methodology?	Yes	Yes	Yes	Yes
2. Is there congruence between the methodology and the question/objectives?	Yes	Unclear	Yes	Yes
3. Is there congruence between the methodology and data collection?	Yes	Yes	Yes	Yes
4. Is there congruence between method and data representation/analysis?	Yes	Yes	Yes	Yes
5. Is there congruence between method and interpretation of results?	Yes	Yes	Yes	Yes
6. Is there a statement theoretically locating the researcher?	No	No	No	No
7. Is the influence of the researcher in the research indicated?	Unclear	Unclear	Unclear	Unclear
8. Are participants and their voices well represented?	Yes	Yes	Yes	Yes
9. Is the research ethical according to current criteria, and is there evidence of ethical approval by an appropriate body?	Yes	Unclear	Yes	Yes
10. Do the conclusions reflect data analysis and interpretation?	Yes	Yes	Yes	Yes

It was evident in the four studies that the influence of researchers during research development was not clear and well delimited as well as the ethical approval of the study dated 1996. It cannot be said which situations permeated the study and made its ethical approval was not clear to readers, however, according to the instrument used, this is an important item and assessed in terms of methodological criticality. Following the methodological order proposed by the institute, dependability, credibility and general analysis assessment by ConQual was evidenced (Chart 2).

**Chart 2:** Synthesis of findings on synthesized findings and their assessments, Maringá, Paraná, Brazil, 2021

Population: relatives of newborns, infants and preschoolers (children from zero to five years).					
**Phenomenon of interest:** the reasons attributed to non-attendance and dropout of follow-up of child growth and development.					
**Context:** child health services, regardless of the level of care.					
**Synthesized findings**	**Study design**	**Dependability**	**Credibility**	**ConQual score**	**Comments**
Family members’ belief that healthy children do not need follow-up of growth and development.	Phenomenological Exploratory Descriptive	High	Moderate	High	Credibility was affected due to the lack of statement locating researchers culturally and their influence on the study.
Daily chores, forgetfulness, lack of prior notice and unforeseen family events to the detriment of child care follow-up.	Phenomenological Exploratory Descriptive	High	Moderate	High	Credibility was affected due to the lack of statement locating researchers culturally and their influence on the study.
Geographical distance and lack of resources as causes of child care drop-out.	Phenomenological Exploratory Descriptive Grounded Theory	High	Moderate	High	Credibility was affected due to the lack of statement locating researchers culturally and their influence on the study.
**Population:** relatives of newborns, infants and preschoolers (children from zero to five years).					
**Phenomenon of interest:** the reasons attributed to non-attendance and dropout of follow-up of child growth and development.					
**Context:** child health services, regardless of the level of care.					
**Synthesized findings**	**Study design**	**Dependability**	**Credibility**	**ConQual score**	**Comments**
The long wait for consultation in the child health service can lead family members to give up children’s follow-up.	Phenomenological Exploratory Descriptive	High	Moderate	High	Credibility was affected due to the lack of statement locating researchers culturally and their influence on the study.

There was a similarity between study designs, since the four articles selected for the final sample fit the category of descriptive and exploratory, probably because this characteristic is frequent within qualitative approaches. It was also noticed that, in the four listed situations, the fact that researchers did not publicly declare their influence within the research caused the credibility of these studies to be questioned. Despite the data obtained, the stage ended with a high score due to unequivocal findings. After that moment, the four articles included in the final sample went through the data extraction process recommended by the JBI and subsequent refinement of information. They are displayed in Chart 3.

**Chart 3:** Synthesis of data extraction, Maringa, Paraná, Brazil, 2021

Title/reference	Year/ country	Design/ number of participants	Interventions	Outcomes	ConQual score quality indicator
*A consulta de puericultura na perspectiva de mães e profissionais de unidades básicas de saúde de Belo* *Horizonte* ^(18)^	2017 Brazil	Descriptive, exploratory, qualitative n = 14	Conducting in-depth interviews with structural analysis of narration.	The main justifications for the absences admitted by mothers were distance, forgetfulness, lack of financial resources, error in consultation date (date had already passed), delay and delay in the consultations, in addition to the lack of attention of the professionals.	High
*O abandono da consulta de enfermagem: uma análise compreensiva do fenômeno* ^(19)^	1996 Brazil	Descriptive, exploratory, qualitative n = 12	Conducting open interviews analyzed in light of the Maurice Merleau-Ponty phenomenological framework.	Absences and dropout were justified by the mothers’ belief that healthy children do not need follow-up. In addition to claiming physical tiredness, long geographic distance to the service. The lack of money to pay for transport, long waiting times for consultations and frequent strikes by the health service were also mentioned as barriers.	High
Why I don’t take my child for a childcare consultation^(20)^	2010 Brazil	Descriptive, exploratory, qualitative n = 16	Conducting semistructured interviews and analyzed based on Minayo’s framework.	The reasons given by the mothers for absences/dropout were forgetfulness, not being informed of consultation day, lack of time, inconvenient consultation times, they see no importance in the consultation, in addition to unforeseen family circumstances.	High
Access to health care for Roma children in central and eastern Europe: findings from a qualitative study in Bulgaria^(21)^	2009 Bulgaria	Descriptive, exploratory, qualitative n = 12	Conducting interviews which were analyzed following the assumptions of theory based on Strauss’ data.	In the reports about non-attendance to health services, poverty, administrative and geographic obstacles were found, in addition to the lack of adaptation to cultural, linguistic and religious specificities.	High

**Figure 2:**
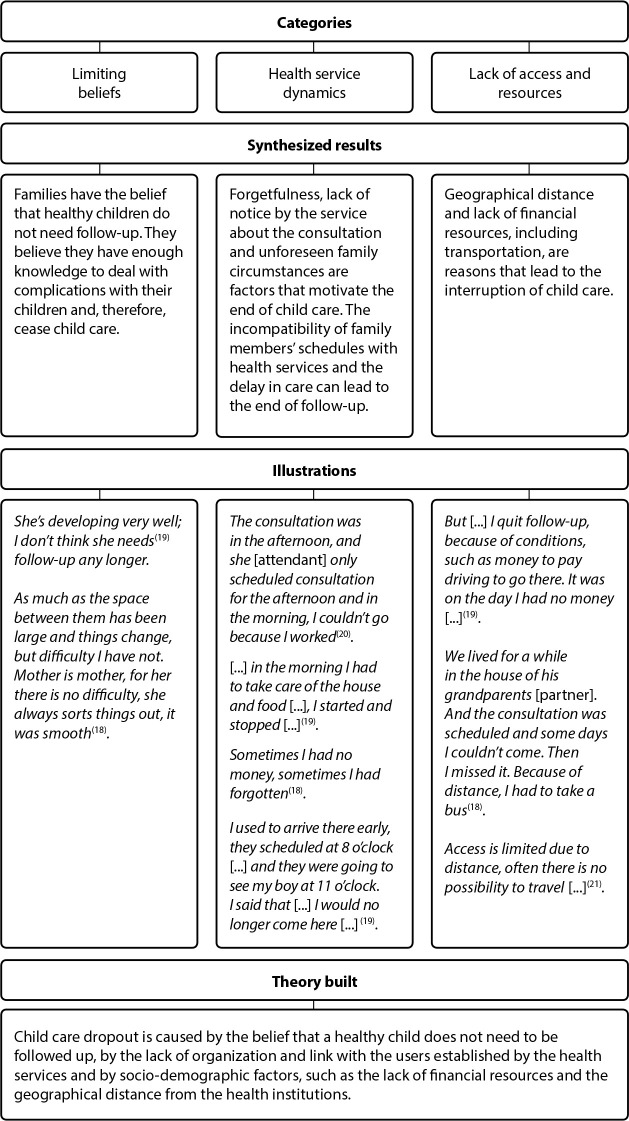
Synthesis of results from the final sample, Maringá, Paraná, Brazil, 2021

Figure 2 indicated that, with the exception of one study, the other three were carried out directly with mothers without fathers’ involvement. All articles had the interview method as a collection method with subsequent content analysis. All works were interested in revealing, in some way, access to health services and how families understood it. Finally, the process of creating the synthesized findings, along with the illustrations that corroborated what was found during the research, were described in Figure 2.

Analyzing Figure 2, it can be stated that the categories that emerged during the analysis process are subdivided into intrinsic and extrinsic factors. Intrinsic factors are directly related to particular knowledge and beliefs regarding children’s health. Extrinsic factors concern the organization of health services, dynamics of care, control of absent children, geographic location, which interferes with access to places of care.

## DISCUSSION

The studies included in the review deal with qualitative research. Only one of them was carried out with a specific population, in this case the gypsies, and the others addressed exclusively mothers. Despite the factors pointed out about the reasons that led to follow-up dropout being repeated among the articles, the scarcity of identified studies demonstrates the need for more in-depth scientific exploration in this context.

The categories that emerged during the analysis process were grouped into three groups, as many of the illustrations are based on similar situations. In order to better discuss the results found, such categories emerged: *Knowledge and beliefs related to childcare: factors that hinder care; Organization and dynamics of work: alert situations regarding child care follow-up*; and *Access to services and resources regarding child health: geographic distance and lack of inputs*.

### Knowledge and beliefs related to childcare: factors that hinder care

Some studies have brought up popular beliefs that a healthy child does not need follow-up and that consultation is not always considered important and pertinent within child follow-up^(19-20)^. In view of this, it is observed that those responsible for the children possibly do not have knowledge about the health condition presented by their child, since child development follow-up consultations, in this context, are offered to follow up the health condition of children who presented problems in the perinatal period or are likely to experience some complication in the first months of life due to prenatal conditions^(19)^.

With regard to child care in the Basic Health Unit, through childcare^(20)^, it is noted that parents do not understand the importance of the consultation carried out by health professionals in the context of health promotion and prevention of diseases and injuries, making it necessary that this perspective be worked on and expanded through health education.

There is scientific evidence that most of the barriers related to attending health services are intrinsically linked to cultural practices and norms^(22)^. However, in this regard, a mixed methods study, carried out with 719 parents, found that one of the factors that corroborate compliance of families with child health services is related to professionals who listen to and respect their particular beliefs and practices and who carry out the guidelines relevant to care, without detracting from previous knowledge, which has been passed down through generations^(23)^.

In this regard, it is suggested the creation of strategies that expand the formation of bonds between family members and health services, looking for spaces in which individual beliefs can be discussed and demystified, while pertinent and updated information is passed on and applied within the child health context.

It was illustrated in the results that the mothers refer to believe that their child is healthy and that they have knowledge about children’s health^(19)^. Families’ lack of understanding about growth and development in childhood can lead to a false interpretation of signs, triggering failures in child care. Working towards health education so that families learn to identify the warning signs, guided during consultations, together with the early detection of health problems, becomes a determining factor in the treatment and recovery of children^(24)^.

The fact that mothers are the population of most studies and the fact that they mentioned that they were unable to attend consultations, claiming that they had other children and even because they had fallen ill, raises the issue of maternal burden. It is known that children’s parents are also responsible for following up their children’s health, however, their presence is not even mentioned.

By taking charge of the processes that permeate relationships within the family environment, mothers end up becoming increasingly burdened and begin to weaken their self-care at the same time that they begin to prioritize the most urgent situations, in order to reduce the demands, in this case, child care dropout. In this context, family support is a variable reported as mitigating stress and overload, in addition to being fundamental for the balance of the social context in which children are^(25)^.

It is worth remembering that missing consultations and the lack of importance given to this conduct means an absence in continuity of care, which may increase the search for emergency care, especially in cases where there is already an established chronic situation. At the same time, defaulters can have other negative impacts, such as delay in detecting problems that will potentially worsen over time^(26)^.

### Organization and dynamics of work: alert situations regarding child care follow-up

Among the studies found, situations directly related to the organization of health services stand out, such as scheduling at inappropriate times, in mothers’ perception, lack of flexibility of schedules for consultations and lack of active search for absentee. Many mothers reported that they forgot about the consultations and had no return from the service due to their absence, in addition to having the perception of little interest shown by the health team in scheduling consultations.

Low compliance and even trust in health professionals and institutions are factors of extreme concern in child health, as the reported situations are linked to dynamics of care and are subject to change, such as the consultation being held at another time. Some health institutions carry out assistance campaigns at alternative times to capture users who work during business hours and are unable to attend face-to-face windows, strategy that can be extended to children, as this population needs the availability of guardians to attend health services^(27)^.

The relationship with care is something broad and multidimensional, since it depends on the degree of correspondence of patients and the fulfillment of expectations of both them and their families, at the same time that it can be influenced by several factors, with children’s physical and emotional condition and the impact that this causes within the family functioning being decisive situations for their development^(28)^.

This relationship between users and professionals, whether they are responsible for children or for their own care, directly interferes with the satisfaction referred to health services. Some studies show that the fact that patients manage to be assisted or only report their complaint within Basic Health Unit (BHU) contributes to their satisfaction with the care received. This fact demonstrates that satisfaction is associated with humanized assistance and active listening and is as important as the structural conditions of the unit itself^(29)^.

The long wait for care is a reality frequently reported in other articles and becomes a reason for dissatisfaction and consequent low compliance by users. They report feeling aggrieved regarding the right of access to health and their perception of quality of care improves when care is provided in a more agile and individualized way^(30)^.

The fact that mothers signaled the lack of interest of nurses who carried out childcare becomes a warning sign, since the lack of credibility and trust in health professionals impairs following up children’s general condition. Studies show that distrust in the professional who provides care leads parents to seek less health care, as well as being related to a higher probability of non-compliance with treatment, frequent visits to emergency rooms and lack of relationships with the health team^(31)^.

Therefore, it is observed that parents’ lack of credibility as legal guardians the children, both in the professional and in the health system, can directly influence the outcomes of child health, especially when considering that this population does not have the ability for self-care, depending exclusively on parents or guardians.

On the other hand, the welcoming, listening and provision for care by the nursing team results in a productive outcome of relationships and the establishment of a bond between those involved^(32)^.

### Access to services and resources regarding child health: geographic distance and lack of inputs

Geographic distance was pointed out in the articles as a factor that hinders access to health services. This barrier directly interferes with dropout rates, since families are unable to reach professionals and, consequently, children are left without care, a fact that is still very common in several regions of the country^(33)^.

The lack of financial resources was also pointed out in articles as a factor that hinders access to health services, causing child care dropout. Unfortunately, within the Brazilian territory we still experience situations in which health institutions are very far from the home, and when the family does not have sufficient financial resources, such as lack of money to pay for public transport, children’s health care is outdated and put in the background^(34)^.

Even in situations where families seek other means of transportation, such as taking a ride, the lack of scheduled consultations is still present, because even in this way it is necessary to pay for fuel and even the people who make this transport service, as in many places health care is entirely paid for by users. Dependence on third parties to reach the institutions, expenses with transport, added to the condition of inherent poverty and the lack of jobs that guarantee a fixed income, means that the money received is directed towards meeting basic needs, with health care being restricted to emergency situations^(21)^.

On the other hand, a study published in another reality showed that, often, families are willing to travel in search of free health services, precisely because they do not have to pay for care^(35)^.

The issue of difficult access for patients interferes both in the outcomes with individuals’ own health and also in service dynamics. The fact of not attending the consultations and, mainly, of not taking children for follow-up, results in risk situations, as many factors are identified during consultations. Moreover, scheduling the consultation and not showing up can lead to loss of financial resources and damage to other patients on waiting lists for assistance^(35-37)^.

It is understood that changing the reality of people who are in a situation of social vulnerability depends on the involvement of many instances within the health services, including managers and professionals, whether physicians or nurses, in order to try to facilitate early and elective care so that actions are carried out aimed at preventing injuries and adequately promoting child health^(37-38)^.

It is noticed that while there are physical and structural barriers that hinder access to health services, there is also a failure in the population’s understanding of the importance of follow-up and taking care of their own health, in the sense of promoting selfcare and care for their own as well as preventing diseases^(38-39)^.

A study carried out with parents, whose focus was the promotion of child health, showed that their understanding of the health care of their children is somewhat superficial, highlighting the need to address more complex issues, regarding the need to promote parental participation in health institutions as well as carrying out scheduled consultations^(36)^.

Therefore, it becomes essential that health authorities follow up absences and seek alternatives for greater compliance with follow-up consultations, both in situations of greater instance, such as geographic and structural barriers, developing in users a sense of responsibility for their own health, in order to ensure greater involvement in care and, consequently, improve followup of child growth and development.

### Study limitations

As a limitation, there is the fact that the number of articles found for analysis is small. It is known that this fact is due to the choice of authors to analyze only articles with a qualitative approach and this interferes with the final number of selected articles. However, it should be noted that all the scientific rigor required of a qualitative systematic review were followed in accordance with JBI assumptions.

### Contributions to nursing, health, or public policies

Health professionals, especially nurses who work in the pediatric area, can benefit from the results found, since the synthesized theory brings up everyday situations that directly influence child care dropout. Once the factors are known, the process of seeking strategies that lead to a decrease in child care dropout rates becomes less laborious and more specific, since prospective research can be based on our results to act in a characteristic way, helping to eliminate the factors that are known to harm child health follow-up.

## FINAL CONSIDERATIONS

This systematic review assessed and synthesized qualitative evidence on the factors that lead to health follow-up dropout of newborns, infants and preschoolers in child health services. The synthesized findings highlight that health follow-up dropout is based on personal knowledge and beliefs, the family routine dynamics and access to services.

The analysis of the findings with the ConQual instrument showed that, despite the credibility of the articles being a factor to be reviewed, since researchers did not make clear their form of participation in the studies, all results have illustrations classified as unequivocal, i.e., they do not leave room for doubts and questions about their veracity, thus classifying them as a high score.

## Data Availability

https://doi.org/10.48331/scielodata.ZELR7S
